# Neuronal and Glial Clocks Underlying Structural Remodeling of Pacemaker Neurons in *Drosophila*

**DOI:** 10.3389/fphys.2017.00918

**Published:** 2017-11-14

**Authors:** Anastasia Herrero, José M. Duhart, Maria F. Ceriani

**Affiliations:** Laboratorio de Genética del Comportamiento, Fundación Instituto Leloir, IIB-BA CONICET, Buenos Aires, Argentina

**Keywords:** circadian remodeling, structural plasticity, LNvs, cell autonomous clocks

## Abstract

A number of years ago we reported that ventral Lateral Neurons (LNvs), which are essential in the control of rest-activity cycles in *Drosophila*, undergo circadian remodeling of their axonal projections. This structural plasticity gives rise to changes in the degree of connectivity, which could provide a means of transmitting time of day information. Thus far, work from different laboratories has shown that circadian remodeling of adult projections relies on activity-dependent and -independent mechanisms. In terms of clock- dependent mechanisms, several neuronal types undergoing circadian remodeling hinted to a differential effect of clock genes; while *per* mutants exhibited poorly developed axonal terminals giving rise to low complexity arbors, *tim* mutants displayed a characteristic hyper branching phenotype, suggesting these genes could be playing additional roles to those ascribed to core clock function. To shed light onto this possibility we altered clock gene levels through RNAi- mediated downregulation and expression of a dominant negative form exclusively in the adult LNvs. These experiments confirmed that the LNv clock is necessary to drive the remodeling process. We next explored the contribution of glia to the structural plasticity of the small LNvs through acute disruption of their internal clock. Interestingly, impaired glial clocks also abolished circadian structural remodeling, without affecting other clock-controlled outputs. Taken together our data shows that both neuronal and glial clocks are recruited to define the architecture of the LNv projections along the day, thus enabling a precise reconfiguration of the circadian network.

## Introduction

Plasticity—the ability to make adaptive changes—is an integral property of the nervous system. There are numerous examples of functional and structural plasticity in invertebrates and vertebrates (Holtmaat and Svoboda, 2009; Bozorgmehr et al., 2013), highlighting its relevance. Plasticity occurs at different scales in time and structure, ranging from milliseconds to hours and from dendritic spines and axonal boutons to entire axonal and dendritic arbors. Structural plasticity contributes to synaptic and circuit function, and it is affected during aging (Barnes, 2001) and disease (Bernardinelli et al., 2014); despite their relevance, the mechanisms underlying structural plasticity, especially large-scale terminal remodeling in the adult brain remains elusive. Over the years, examples of structural remodeling of neuronal terminals taking place along the day have accumulated (Pyza and Meinertzhagen, 1999; Mehnert et al., 2007; Becquet et al., 2008; Fernández et al., 2008; Appelbaum et al., 2010), and have been shown to depend on an intact circadian clock (Fernández et al., 2008).

The circadian clock is conserved throughout the animal kingdom. In *Drosophila*, 150 neurons in the adult brain support a circadian pacemaker. This molecular clock depends on the activity of the transcription factors CLOCK (CLK) and CYCLE (CYC), which drive circadian oscillations by promoting rhythmic transcription of several key genes, including *period* (*per*), *timeless* (*tim*), and *clockwork orange* (*cwo)*, which repress CLK-CYC-mediated transcription (Ozkaya and Rosato, 2012). The coordinated operation of the circadian network is necessary for the adapted responses to synchronizing environmental stimuli. Clock neurons are anatomically clustered in distinct groups: small and large ventral-lateral (s-LNvs, l-LNvs, and the fifth s-LNv), the dorsal-lateral (LNds), the lateral posterior (LPNs) and three subgroups of dorsal neurons (DNs1-3). Only the LNvs express a neuropeptide called PIGMENT DISPERSING FACTOR (PDF), which plays a major role in the synchronization of the circadian network. PDF is essential for normal circadian activity patterns in light: dark cycles (LD) and for persistent circadian rhythms under constant free running conditions (DD). In fact, PDF synchronizes the phase of the sLNvs and DN1s, while slows down the pace and increases the amplitude of the LNds and the PDF negative 5th sLNv (Lin et al., 2004; Lear et al., 2009; Yoshii et al., 2009; Im et al., 2011).

PDF immunoreactivity changes throughout the day in the sLNv axonal termini, indicating that its regulation is under clock control. In addition, the sLNv axonal terminals exhibit a higher degree of arborization during the day and a reduced complexity at night, accompanying the changes in PDF levels. This phenomenon, called circadian structural plasticity, is lost in *per*^01^ and *tim*^01^ mutants, indicating that it depends on a functional clock, although substantial structural differences are observed (Fernández et al., 2008).

Glial cells have a critical role in plasticity and synaptic transmission. Recent studies in *Drosophila* have implicated glial cells in the regulation of neuronal excitability, vision, circadian behavior, sleep, behavioral sensitivity to drugs, and olfaction (Borycz et al., 2002; Bainton et al., 2005; Stuart et al., 2007; Suh and Jackson, 2007; Ng et al., 2011; Seugnet et al., 2011; Melom and Littleton, 2013; Chaturvedi et al., 2014; Liu et al., 2014; Chen et al., 2015). Despite little is known about *Drosophila* circadian gliotransmitters, there is vast evidence of their relevance in circadian rhythmicity (Ng et al., 2011; Ng and Jackson, 2015). Several studies have reported rhythmic expression of clock proteins and other neural proteins (e.g., PER, TIM, Ebony) in glial cells of the adult brain (Suh and Jackson, 2007), although the importance of glial clocks in circadian outputs has not been extensively studied yet, prompting us to analyze its relevance in structural plasticity.

Given the implications to the circadian network of an active sLNv terminal remodeling, i.e., daily changes in connectivity (Gorostiza et al., 2014), we inquired whether affecting the molecular clock, particularly in the adult LNvs, would abolish circadian plasticity. To this end we deregulated specific clock genes and analyzed the impact of these genetic interventions on structural remodeling; interestingly, despite altering different clock genes similarly affected molecular oscillations, the terminals adopted a different configuration, suggesting that additional mechanisms are recruited. We next addressed the possibility that the clock in glial cells actively contributes to the structural plasticity of sLNv terminals, and uncovered that adult-specific impairment of their molecular clock also disrupts circadian remodeling. Thus, both, the molecular clock in LNvs and glia are necessary for sustaining this unusual form of plasticity.

## Materials and methods

### Fly rearing and strains

Flies were grown and maintained at 25°C in vials containing standard cornmeal medium under 12:12 h LD cycles. For adult specific induction either the GeneSwitch or TARGET systems were employed (McGuire et al., 2004). GeneSwitch expression was induced transferring 2 day-old flies to vials containing food supplemented with RU486 (mifepristone, Sigma, USA) in 80% ethanol to a final concentration of 200 μg/ml, or with the same amount of ethanol (vehicle) in control treatments. Adult-specific termosensitive Gal4 expression was induced transferring flies raised at 23°C during development to 30°C for 48 h. The *pdf*-GeneSwitch (*pdf*-GS) line was generated in our laboratory (Depetris-Chauvin et al., 2011); Stocks UAS-*cyc*^*DN*^ (#36317, Tanoue et al., 2004), UAS-*tim*^*RNAi*^*I* (#29583), UAS-*per*^*RNAi*^ (#40878 and #31285, I and II, respectively), *pdf*-Gal4 (#6900), *repo*-Gal4 (#7415) were obtained from the Bloomington Stock Center. The UAS-*tim*^*RNAi*^ *II* (#2886) stock was obtained from the Vienna RNAi Stock Center. *pdf*-dsRed was generously provided by J. Blau.

### Adult locomotor activity

For locomotor activity experiments adult male flies were entrained for 3 days in 12:12 LD cycles at 25°C and then transferred to constant darkness (DD) at 25°C. Males were placed in glass tubes containing standard food and monitored for activity with infrared detectors and an automated data collection system (TriKinetics, Waltham, MA). Activity was monitored for 14 days (4 in LD and 9–10 in DD). Period, FFT and rhythmicity in DD were estimated using ClockLab software (Actimetrics, Evanston, IL) as previously described (Ceriani et al., 2002; Depetris-Chauvin et al., 2011).

### Immunohistochemistry and image acquisition

Adult fly heads were fixed with 4% p-formaldehyde (pH 7.5) for 30–40 min at room temperature. Brains were dissected and rinsed four times in PT buffer (PBS with 0.1% Triton X-100) for 30 min. Samples were blocked in 7% normal goat serum (in PT) for 1 h, and incubated with primary antibodies at room temperature for 2 days. The primary antibodies employed were chicken anti-GFP 1:500 (Aves Labs, Inc, USA), rabbit anti-DsRed 1:500 (Clontech, USA) and homemade rat anti-*Drosophila*-PDF 1:500 (Depetris-Chauvin et al., 2011). Samples were washed 4 x 15 min in PT, and incubated with secondary antibody at 1:250 for 2 h at room temperature. Secondary antibodies were washed 4 x 15 min in PT and mounted in Vectashield antifade mounting medium (Vector Laboratories, USA). The secondary antibodies used were Cy2-conjugated donkey anti-rabbit, Alexa Fluor 647-conjugated AffiniPure donkey anti-rat and Cy3-conjugated AffiniPure donkey anti-rabbit (Jackson ImmunoResearch, USA). Images were taken on a Zeiss LSM 710 confocal microscope.

### Structural plasticity analysis and PDF immunoreactivity

Images were taken with a 40× objective and an optical zoom of 2×. CD8GFP signal was adjusted to threshold levels generating a selection that delimits the area of sLNv axonal terminals. This selection was then applied to the PDF channel and mean intensity was measured. For the analysis of PDF immunoreactivity all pictures were taken employing the same confocal settings and quantification was performed using Image J software (downloaded from http://rsbweb.nih.gov/ij/). Structural plasticity was analyzed by Scholl analysis, as reported (Fernández et al., 2008). In all cases the analysis was performed blind.

### Quantitative real-time PCR

Total RNA isolation from fly head extracts was performed using Trizol (Invitrogen, Carlsbad, CA). Superscript III was used for reverse transcription (ThermoFisher Scientific, USA) and FastStart Universal SYBR Green Master (Roche) was used for quantitative real-time PCR following manufacturer's instructions. The real-time assays were conducted in a Stratagene Mx3000P QPCR System (La Jolla, CA) using SYBR green as the detection system and ROX as the reference dye. The primers were designed using Primer3 (available online at http://frodo.wi.mit.edu/primer3/). mRNA levels were assessed from three independent RNA extractions and two technical replicates were performed on each sample. Only primer pairs with efficiency between 90 and 110% were used. The following primers were employed, to detect *rpl49* (fw 5′GAACAAGAAGGCCCATCGTA3′; rev 5′AGTAACAGGCTTRGGCTTGC3′); *per* (for 5′GACCGAATCCCTGCTCAATAA3′; rev 5′GGACTTCTTGCTCTTCTCACC3′); *tim* (fw 5′GGTAAACGGATCGCACTTCTCG3′; rev 5′AAGAGACATTGTCGCTGTTTAAT3′); *dClk* (fw 5′CAGAGTCAGTTGCAGGATCAA3′; rev 5′GCAGATATGTGTAGCGGGATAG3′); *cyc* (fw 5′TGGACAATCACCCGAACATAC3′; rev 5′CTGAGGCAGGAAACCAATCA3′).

### Data analysis and statistics

Statistical analyses were performed with the InfoStat package version 2009 (Grupo InfoStat, FCA, Universidad Nacional de Córdoba, Argentina). In all graphs, experimental groups with different letters indicate statistically significant differences, with a *p* < 0.05. Validation of RNAi lines was tested with a Student's *t*-test. Clock gene oscillation under different clock modulations was analyzed by One-way ANOVA within each clock gene, followed by Tukey's *post-hoc* test. Effects on structural plasticity were analyzed by Two-way ANOVA, followed by Tukey's *post-hoc* test. Effects on PDF levels were analyzed by Kruskal–Wallis One way ANOVA, followed by Conover's *post-hoc* test. Number of flies or brains in each experiment is referred as *n*, and the number of experiments is referred as *N*, and was used for statistical analysis.

## Results

### Disrupting the molecular clock in the adult LNvs impairs locomotor rhythmicity

To address the possibility that the circadian remodeling of sLNv terminals exclusively depends on their own molecular clock we took advantage of the spatially restricted Gal4/UAS system to alter clock protein levels exclusively in the LNvs. To monitor the efficiency of the different strategies and potentially uncover a differential effect, we first analyzed locomotor activity patterns in flies in which different clock genes were constitutively deregulated through RNAi-mediated silencing (*per, tim*), or through the expression of a dominant-negative CYC version (CYC^DN^, Tanoue et al., 2004).

**Figure 1:**
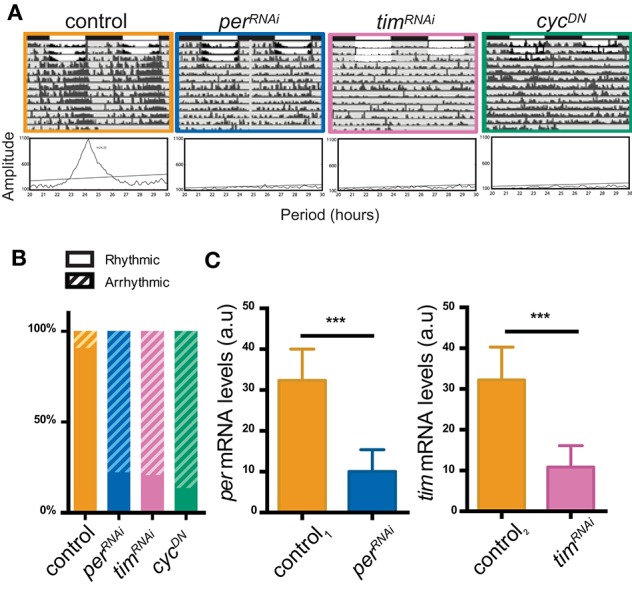
Impaired LNv clocks result in disrupted locomotor activity patterns. **(A)** Representative double-plotted actograms and their respective periodograms of the different genotypes: UAS-*dcr2*, UAS-*per*^*RNAi*^*I* (#40878), UAS-*tim*^*RNAi*^*I* (#29583), and UAS-*cyc*^*DN*^ under the control of *pdf-*Gal4. Locomotor activity of individual flies was recorded for 4 days under 12:12 LD cycles and then transferred to DD (gray area) for 9 additional days. In the actograms, white bars represent day, black bars represent night. For every fly actogram, periodograms of the free-running rhythm in DD are shown (bottom). Rhythmic behavior of a typical male control is shown (orange). *per*^*RNAi*^ (in blue), *tim*^*RNAi*^ (in pink), and *cyc*^*DN*^ (in green) expressing animals are shown. All genetic manipulations gave rise to a largely arrhythmic locomotor behavior. **(B)** Percentage of rhythmicity. Data represents at least three independent experiments; over 44 flies were analyzed. **(C)** Validation of the effectiveness of the RNAis employed. Both UAS-*per*^*RNAi*^ and UAS-*tim*^*RNAi*^ were expressed under *tim*-Gal4. Levels are normalized to *rpl49*. Both *tim and per* mRNA levels are reduced compared to their respective control. Student's *t*-test showed a significant difference in levels of expression. Triple asterisks (^***^) indicate significant differences with *p* < 0.001. Three independent experiments were performed.

Control (*pdf-*Gal4>+) flies showed a clear rhythmic pattern in the presence of synchronizing cues (LD cycles) as well as in DD (Figure 1A). In DD this rhythm has a period of around 24 h and flies consolidate their activity across the subjective day (Table 1). Downregulating *per* and *tim* mRNA levels in the LNvs (through *pdf*-Gal4) employing different RNAi lines triggered significant effects on the patterns of locomotor activity, mostly a ~80% decrease in the percentage of rhythmicity (Figure 1B); surprisingly, period length was not different from the control (Supplementary Figure 1 and Table 1). We next measured steady state levels of *per* and *tim* mRNA by quantitative real-time PCR in total RNA extracts from wild type (*tim-*Gal4,*dcr2*>+) and flies expressing specific RNAis (*per*^*RNAi*^ and *tim*^*RNAi*^) at the peak of their endogenous levels (CT14, CT stands for circadian time, and refers to the hours passed since the last day to night transition). Significant differences were observed, with a decrease to about 30% compared to wild type levels both in the case of *per* and *tim* mRNA (Figure 1C). To overcome potential unspecific effects of the knockdown strategy, we employed a second RNAi line that showed similar results (Supplementary Figure 1). Thus, both RNAis are efficient to downregulate clock protein levels and affect clock outputs (i.e., behavioral rhythmicity).

**Table 1:** Detailed circadian parameters for all the behavioral experiments performed.

**Genotype**	**%R ± SEM**	**Tau ± SEM**	**FFT ± SEM**	**Power ± SEM**	***N***	***n***
*pdf*GAL4> +	90.69 ± 3.44^A^	24.26 ± 0.4	0.035 ± 0.009	725.37 ± 353.4	3	66
*pdf*GAL4>*per^*RNAi*^ I*	22.31 ± 9.78^B^	23.94 ± 0.12	0.023 ± 0.008	247.42 ± 143.71	3	70
*pdfGAL4>tim^*RNAi*^ I*	20.72 ± 15.99^B^	23.50 ± 0.24	0.013 ± 0.003	347.40 ± 126.48	3	61
*pdf*GAL4>*cyc^*DN*^*	13.73 ± 3.34^B^	23.67 ± 0.09	0.018 ± 0.004	175.60 ± 53.67	3	46
*pdfGAL4>per^*RNAi*^ II*	43.54 ± 13.91^AB^	23.72 ± 0.09	0.024 ± 0.007	336.92 ± 189.72	3	73
*pdfGAL4>tim^*RNAi*^ II*	46.26 ± 16.75^AB^	24.33 ± 0.44	0.022 ± 0.007	506.65 ± 155.42	3	78

*The analysis included the assessment of period (Tau) and different measurements of rhythm strength, such as FFT, power, and percentage of rhythmicity (%R). Average ± S.E.M. of different clock deregulating genotypes along with the control is shown. Different letters indicate statistically significant differences with a p < 0.05 (One-way ANOVA with a Tukey post-hoc test). N indicates the number of experiments, which was used for statistical analysis. n indicates the number of animals tested. per^RNAi^ I (Bloomington Stock Center: #40878) and tim^RNAi^ I (Bloomington Stock Center: #29583) refers to the RNAis used in the main figures. The ones indicated in gray correspond to the RNAi lines shown in Supplementary Figure 1, as follows: per^RNAi^ II (Bloomington Stock Center: #31285) and tim^RNAi^ II (VDRC Stock Center: #2886)*.

In addition, we took advantage of a well-characterized dominant negative variant of CYC, called CYC^DN^ (Tanoue et al., 2004) to block CLK/CYC activated transcription. As previously reported, expressing *cyc*^*DN*^ in the LNvs produced a drastic reduction of behavioral rhythmicity (Figures 1A,B; see Table 1 for an in depth analysis of behavioral parameters) down to about 14%, suggesting this strategy is a very efficient one to impair clock function (Tanoue et al., 2004). Taken together, these experiments support the relevance of LNvs in the control of rhythmic rest-activity cycles since deregulating different core clock genes in a cluster-specific fashion significantly impacts the consolidation of rhythmic locomotor behavior.

### Modulation of different clock components leads to dampened oscillations

Rhythmicity of clock-controlled outputs depends on the precise regulation of the cell autonomous molecular clock. Since affecting different clock protein levels in the LNvs altered behavioral patterns to a different degree we assessed the state of the molecular clock after long term deregulation of each specific clock protein. To this end we examined *per, tim, dClk*, and *cyc* mRNA levels at two timepoints during the day, CT2 and CT14, upon deregulation of the different genes using the pan-circadian driver *tim-*Gal4.

**Figure 2:**
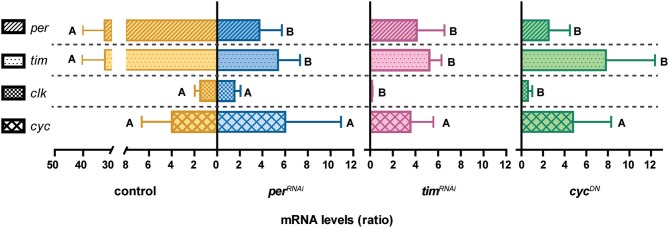
Different clock components trigger dampened molecular oscillations. For each gene, the ratio describing higher/lower mRNA levels is plotted (that is, CT14/CT2 for *per, tim* and *cyc*; and CT2/CT14 for *clk*). Levels are normalized to the reference gene *rpl49*. Statistical analysis was performed comparing individual transcript levels (indicated by a dashed line). The genotypes analyzed are as follows: control (orange), *per*^*RNAi*^*I* (blue), *tim*^*RNAi*^*I* (pink), and *cyc*^*DN*^ (green), under *tim-*Gal4. Different letters indicate statistically significant differences with a *p* < 0.05 (One-way ANOVA with a Tukey *post-hoc* test). Three independent experiments were performed.

Interestingly, we found that the amplitude of the peak/trough oscillation of *per* or *tim* transcript levels was greatly reduced in the conditions tested regardless of the clock protein targeted, that is, as a result of impairing repression or activation (Supplementary Figure 2 and Figure 2). As expected (Lerner et al., 2015), *dClk* mRNA levels were low compared to those of *per* and *tim* in controls, which could partially account for the difficulty in determining a precise change in peak/trough amplitude (only two-fold in this set of measurements for the controls). Accordingly, a clear effect on the amplitude of *dClk* oscillations was detectable in *tim*^*RNAi*^ and *cyc*^*DN*^. In addition, despite *cyc* levels were originally reported not to cycle by northern blot (Rutila et al., 1998), qPCR analysis showed a shallow (three-fold) cycling in endogenous *cyc* levels in controls (higher at CT14 than CT2), that were not significantly affected in any of the combinations analyzed (Supplementary Figure 2).

In sum these results highlight that deregulation of different clock components results in a dampened molecular clock, regardless of the affected process (that is, impaired CLK/CYC mediated transcription or its repression), and confirm that any of these genetic interventions are useful to assess the relevance of the cell autonomous clock on a particular output, i.e., the control of the structural plasticity of the sLNv terminals.

### Circadian structural plasticity is differentially altered by clock genes

A number of years ago we reported morphological changes of the axonal terminals of the sLNvs across the day and showed that this phenomenon is under the control of the circadian clock since it is abolished in *per*^01^ and *tim*^01^ mutants (Fernández et al., 2008). However, despite circadian changes in the complexity of the sLNv axonal arbor was abrogated in both null mutants, the overall morphology of the terminals was quite distinct, suggesting PER and TIM could be playing additional “non-circadian” roles. Thus, we set out to examine the impact of altering different molecular clock components specifically in the LNvs on the architecture of the dorsal terminals exclusively in the adult brain to avoid potential developmental effects.

**Figure 3:**
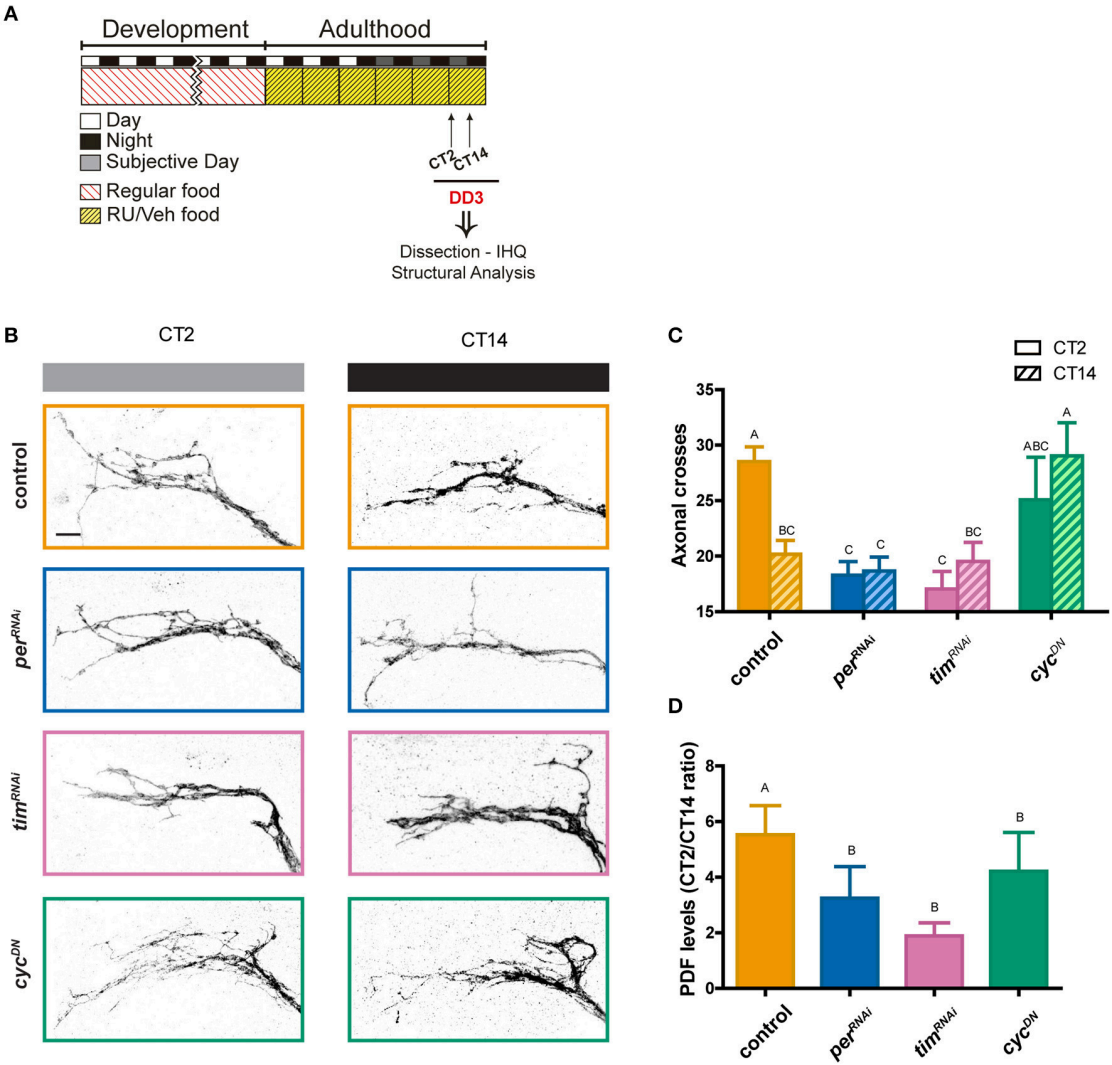
Circadian structural plasticity is differentially altered by clock genes. **(A)** Schematic diagram illustrating the standard protocol. “Veh” and “RU” stand for “vehicle” and “RU486” containing fly food. **(B)** Representative confocal images of GFP immunoreactivity at the dorsal protocerebrum at the early subjective day (CT2, gray bar) and early subjective night (CT14, black bar) during the 3rd day of constant darkness (DD3). **(C)** Quantitation of total axonal crosses. Control flies display circadian structural remodeling of axonal terminals while animals with a deregulated clock show no differences across the day. Data represents the average of 3 experiments; a minimum of 27 brains were analyzed per CT/genotype. Different letters indicate statistically different treatments with a *p* < 0.05 (Two-way ANOVA with a Tukey *post-hoc* test, *n* = 8–10, *N* = 3). Controls in vehicle are not different from controls in RU containing food (Depetris-Chauvin et al., 2011). **(D)** Quantitation of PDF immunoreactivity at the dorsal protocerebrum at CT2 and CT14 on DD3. For a more direct comparison, PDF levels are shown as the ratio between CT2 and CT14. Control flies (orange), exhibit circadian oscillation of PDF levels, while different clock deregulation genotypes were significantly different from the control. Different letters indicate statistical differences with a *p* < 0.05 (Kruskal–Wallis One-way ANOVA, followed by Conover *Post-hoc* test, *n* = 8–10, *N* = 3).

We took advantage of an inducible Gal4 version termed GeneSwitch with restricted expression to the LNv neurons (*pdf*-GS, Depetris-Chauvin et al., 2011), combined with a membrane-tethered version of GFP (CD8GFP) to describe the complexity of the axonal arborizations, along with *per*^*RNAi*^, *tim*^*RNAi*^, or *cyc*^*DN*^ to address the role of the LNv clock in structural plasticity. Flies transferred to RU486-containing food 2 days after eclosion were dissected at CT2 and CT14 on DD3 (Figure 3A). As previously reported, the overall structure of the dorsal terminals is more complex in the morning and less arborized at night time in controls (Figures 3B,C, shown in orange; Fernández et al., 2008; Gorostiza et al., 2014). Interestingly, affecting the negative elements of the molecular clock, *per* (shown in blue) and *tim* (in pink), the complexity was significantly reduced from that displayed by controls in the subjective morning (Figures 3B,C), and resembled the nighttime configuration of control terminals. On the other hand, expressing *cyc*^*DN*^ (in green) gave rise to maximally spread axonal termini throughout the day; in fact, the architecture of the termini (to the level described through confocal microscopy) was different from that of controls at any timepoint; specifically, the number of higher order neurites (ramifications of primary and secondary processes) was clearly increased compared to controls, suggesting that actively impairing CYC function in the adult triggers clear morphological defects, beyond those anticipated from affecting the endogenous molecular clock.

PDF immunoreactivity in the axonal terminals at the dorsal protocerebrum has been shown to oscillate in a circadian fashion both under LD and DD conditions (Park et al., 2000); remarkably, this cycling is blocked in mutants with impaired clock function (Park et al., 2000). Immunohistochemistry analysis on whole-mount adult brains dissected at times when PDF levels peak and reach a trough was examined. PDF levels were assessed at CT2 and CT14 upon adult- specific expression of either *per*^*RNAi*^, *tim*^*RNAi*^, or *cyc*^*DN*^ along with controls. Control *pdf*-GS > CD8GFP flies (orange) in the presence of RU486 exhibited a significant difference in PDF immunoreactivity between these two time points (Figure 3D). In contrast, PDF immunoreactivity at the sLNv dorsal terminals in every experimental condition was significantly different to controls. Such difference was more pronounced when expressing *per*^*RNAi*^ (blue) or *tim*^*RNAi*^ (pink), in which the amplitude of the oscillation is markedly reduced, even when compared to *cyc*^*DN*^ (green).

In sum, these experiments demonstrate that circadian remodeling of the sLNv terminals is driven by the LNv molecular clock. Furthermore, affecting the positive and negative elements of the feedback loop triggered a distinctive “architecture” of the axonal termini. Under these conditions, not only structural plasticity but also PDF levels are altered, indicating that both outputs are dependent on the correct operation of the LNv molecular oscillator.

### Glial clocks also contributes to circadian remodeling of the sLNv terminals

Having demonstrated that the LNv clock is necessary for the remodeling of the sLNv termini, we wondered whether additional clocks could contribute to this phenomenon. One evident candidate is the one in glial cells, which plays a role in rhythmic locomotor behavior (Ng et al., 2011); in addition, it is well established that astrocytes modulate the activities of many different neuronal synapses, further strengthening this possibility. Flies expressing *cyc*^*DN*^, in our hands the most effective means to block CLK/CYC activated transcription (Figures 1, 2), was employed to acutely interrupt the glial clock in the adult brain.

**Figure 4:**
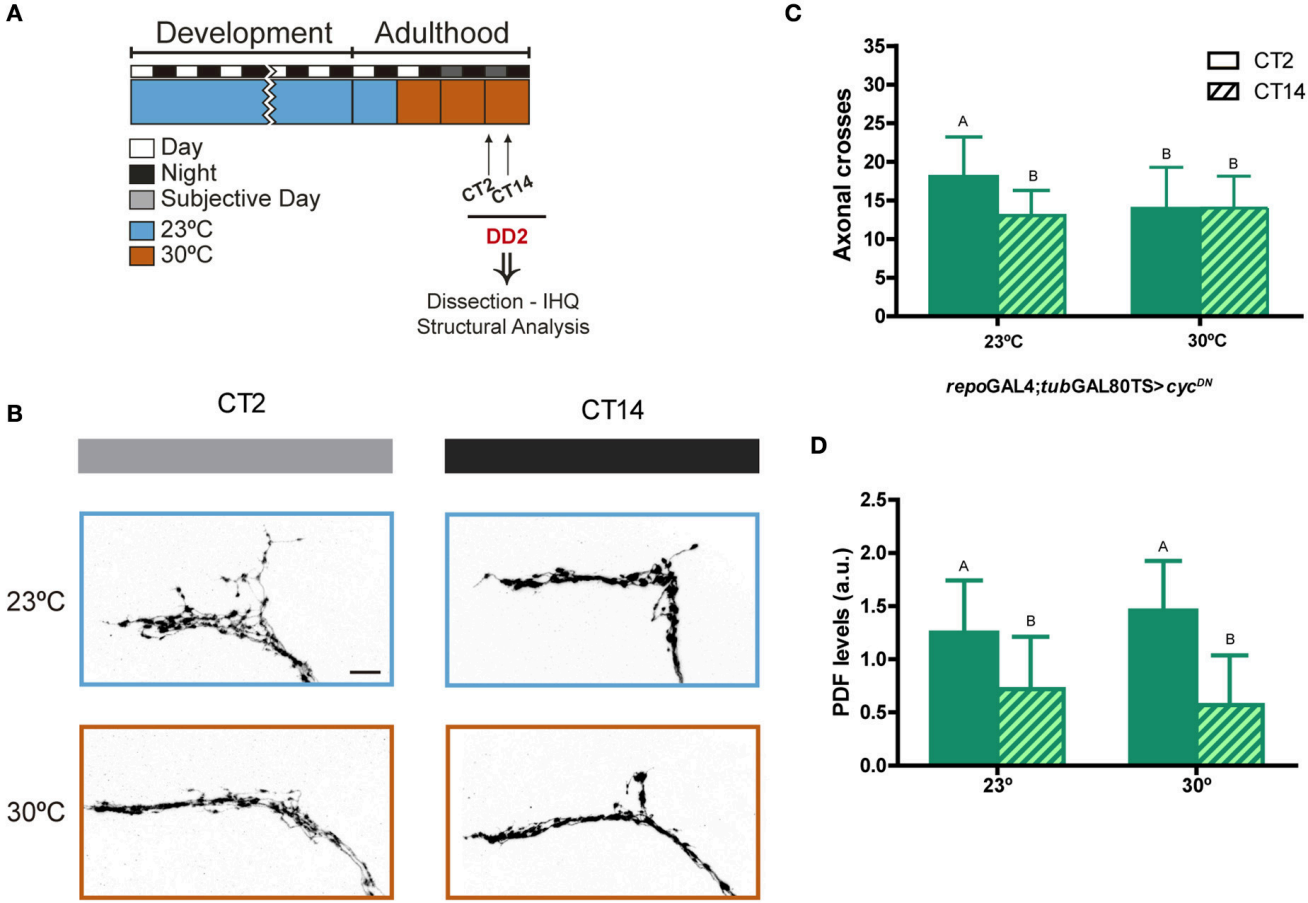
Clocks in glia are required for circadian remodeling of neuronal terminals. **(A)** Schematic diagram illustrating the standard protocol; the restrictive condition is highlighted in light-blue (23°C), and the permissive condition is shown in orange (30°C). **(B)** Representative confocal images of dsRed immunoreactivity at the dorsal protocerebrum of flies containing UAS-*cyc*^DN^ under *repo-*Gal4;*tub-*Gal80^TS^; *pdf* RED enables visualization of the axonal terminals. Brains were dissected at the early subjective day (CT2, gray bar) and early subjective night (CT14, black bar) during the 2nd day of constant darkness (DD2), which corresponds to the 3rd day of permissive condition (30°C). Control flies (always maintained at 23°C) are indicated in light-blue. **(C)** Quantitation of total axonal crosses of *repo-*Gal4;*tub-*Gal80^TS^ > *cyc*^*DN*^. Control flies (kept at 23°C) display circadian structural remodeling of axonal terminals while animals induced at 30°C show no differences along the day. Data represents the average of 3 experiments; a minimum of 27 brains were analyzed per CT/genotype. Different letters indicate statistical differences with a *p* < 0.05 (Two-way ANOVA with a Tukey *post-hoc* test, *n* = 8–10, *N* = 3). **(D)** Quantitation of PDF immunoreactivity at the dorsal protocerebrum from brains dissected at CT2 and CT14 on DD3. Control flies (23°C), exhibit circadian oscillation of PDF levels; those expressing *cyc*^DN^ at 30°C were not significantly different from controls. Same letters indicate no statistically different conditions (*p* > 0.05) (Kruskal–Wallis One-way ANOVA, followed by a Conover *post-hoc* test, *n* = 8–10, *N* = 2). Data represents the average of 2 experiments; a minimum of 16 brains were analyzed per CT/genotype.

At the restrictive temperature (23°C, Figure 4A), where no expression of the dominant negative CYC is achieved, *repo-*Gal4;*tub-*Gal80^TS^ >*cyc*^*DN*^ flies exhibited the expected remodeling of the sLNv terminals, more elaborated during the subjective day than at night (Figures 4B,C). In contrast, disrupting the clock in glia led to the absence of circadian remodeling, resulting in a minimally spread arbor, reminiscent of controls at night (Figures 4B,C). Controls shifted to the permissive temperature still displayed circadian remodeling (Supplementary Figure 3). These observations bring further support to the notion that glial clocks play an active role regulating circadian clock outputs, and more specifically, are essential for circadian structural plasticity.

We also explored PDF immunoreactivity in the dorsal protocerebrum, given its established relevance to this form of plasticity (Depetris-Chauvin et al., 2014). Surprisingly, we found that PDF levels still change between the subjective day and night when the clock in glia is impaired; in fact, under these conditions PDF levels were not different from controls (Figure 4D), providing further evidence that these two clock outputs (PDF levels and structural remodeling) can be uncoupled (Depetris-Chauvin et al., 2011).

Together these results indicate that circadian structural plasticity of the sLNvs depends not only on its own molecular clock, but also that glial clocks actively contribute to this form of plasticity.

## Discussion

A number of years ago we discovered that in wild type flies there are conspicuous structural changes in neurons that are key to the control of circadian locomotor activity (the sLNvs), which undergo remodeling of their axonal arborizations on daily basis (Fernández et al., 2008). Those initial observations led to additional discoveries, namely, that circadian remodeling involves changes in the number of synapses and connectivity, and concomitantly, that sLNvs neurons contact different postsynaptic targets across the day (Gorostiza et al., 2014); thus, structural plasticity results in changes in the strength of the communication between circadian clusters that could contribute to seasonal adaptation (Gorostiza et al., 2014; Petsakou et al., 2015). Circadian structural remodeling has been observed in clock brain structures and in other brain regions receiving input from the circadian clock (Bosler et al., 2015); interestingly, it has been shown to coexist with circadian changes in neuronal excitability and synaptic efficacy within and outside clock structures, but the precise relationship between these two forms of plasticity remains poorly understood (Frank and Cantera, 2014).

### Cell autonomous mechanisms underlie continuous remodeling of neuronal terminals

Rhythmic changes in neuronal morphology reported thus far in *Drosophila* include those in axonal caliber, branching complexity, synaptic vesicles and synapse numbers. One of the structures undergoing daily and circadian remodeling is the visual system that shows changes in the number of synaptic contacts as well as in the morphology of neurons and glial cells (Pyza and Meinertzhagen, 1999; Weber et al., 2009; Damulewicz et al., 2013; Gorska-Andrzejak et al., 2013); likewise, cyclical changes in neuronal morphology are exhibited by motor neurons in the adult (Mehnert et al., 2007; Mehnert and Cantera, 2008; Ruiz et al., 2010, 2013), as well as by interneurons in the central brain (Fernández et al., 2008).

In terms of the underlying molecular processes that trigger structural changes not much is known; activity dependent and independent mechanisms appear to be recruited to drive terminal remodeling in the central brain (Depetris-Chauvin et al., 2011; Sivachenko et al., 2013), associated to changes in the degree of fasciculation (Sivachenko et al., 2013), pruning (Depetris-Chauvin et al., 2014), and actin cytoskeleton remodeling (Petsakou et al., 2015), although the timing of these events has not been explored in any length.

As it is the case for many clock-controlled outputs, it was expected that at least part of the molecules responsible for orchestrating active structural remodeling show circadian modulation of gene expression, protein stability and/or activity. Consequently, circadian remodeling would be directly controlled by a cell-autonomous circadian clock, i.e., the one operating in those specific neurons, a possibility that had not been specifically examined yet. Thus, to examine whether the LNv molecular clock is necessary to drive structural remodeling we resorted to different genetic strategies to obliterate molecular oscillations. Surprisingly, while adult-specific downregulation of the repressors of the molecular clock (through the expression of *per*^*RNAi*^ and *tim*^*RNAi*^) resulted in less complex arborization patterns, impairing CLK/CYC mediated transcriptional activation (through the expression of *cyc*^*DN*^) correlated with maximally spread terminals, despite an overall similar effect on clock genes at the mRNA level. Closer inspection of the architecture of the arborizations suggests that additional phenomena are also affected; particularly in the case of CYC^DN^ expression, not only the terminals are complex throughout the day as controls exhibit in the early (subjective) morning, but also membrane integrity appears severely disrupted unlike neuronal terminals in controls. Additional experiments are required to understand this process in full; one obvious candidate that could mediate the altered morphology is the circadian modulation of actin dynamics (Petsakou et al., 2015) that directly impacts on structural integrity and plasticity of neurons and their synapses. These results underscore that the circadian clock not only drives circadian remodeling but it could additionally play an active role in maintaining neuronal shape (Mehnert and Cantera, 2011).

### Distinct long term and acute effects derived from clock disruption

While addressing the circadian nature of the remodeling phenomenon in different structures it became clear that loss-of-function mutations in *per* and *tim* not only abolish circadian remodeling but also trigger abnormal branching, which in turn would suggest that circadian plasticity plays a role in maintaining normal morphology (Mehnert and Cantera, 2011). Incidentally, *per* and *tim* null mutants showed quite distinct phenotypes in the architecture of the neuronal terminals (Mehnert et al., 2007; Fernández et al., 2008), suggesting that both proteins could play additional roles than those limited to the circadian clock. To delve further into this possibility, we downregulated *per* or *tim* levels in the post-developmental brain, once the whole circadian network was established and well connected. Interestingly, adult-specific downregulation of either gene resulted in a similar structure, which at all times resembled the less complex arborization pattern exhibited by controls at night. These results reinforce the notion that the structural differences associated to loss of function mutations could uncover additional processes in which these proteins participate during early development or during establishment of the circadian circuitry.

### The contribution of glial clocks to LNv structural plasticity

Over the years it was established that glia, and particularly neuron-glia communications, plays an active role in the control of rhythmic outputs, affecting PDF immunoreactivity at the dorsal terminals and concomitantly, rhythmic behavior (Suh and Jackson, 2007; Ng et al., 2011; Ng and Jackson, 2015). Surprisingly, affecting glial clocks *per se* did not impact on rhythmic patterns of locomotor activity, at least upon chronic downregulation of PER levels (Ng et al., 2011).

In the visual system circadian remodeling of neuronal terminals is likely driven by different circadian oscillators, and takes place in photoreceptor cells, the most abundant peripheral oscillator in the fly head, but also in non- clock cells such as the L1 and L2 monopolar neurons in the lamina (Weber et al., 2009). In the latter, circadian input driving remodeling likely derives from the photoreceptors, the PDF+ central clock neurons, as well as the surrounding glia (reviewed in Gorska-Andrzejak, 2013). Structural changes also correlate with changes in the abundance of a marker of presynaptic active zones (Bruchpilot, BRP; Gorska-Andrzejak et al., 2013). Interestingly, it was reported that blocking clock function in glia alters, though it does not obliterate, the daily changes in BRP accumulation in the lamina cartridges (Gorska-Andrzejak et al., 2013). In the house fly, glial cells change in size in the opposite phase compared to neurons, and remodeling is affected either when perturbing glial metabolism or, more dramatically, glial communication (Pyza and Gorska-Andrzejak, 2004).

To begin to assess whether clocks in glia would contribute to the structural remodeling of the LNv projections, panglial CYC^DN^ expression was restricted to the adult. Interestingly, acute (for 2 days) disruption of glial clocks completely abolished circadian plasticity, underscoring their active contribution to the remodeling process. However, under those conditions, PDF immunoreactivity at the dorsal terminals exhibited no differences compared to controls, unexpectedly uncoupling both clock outputs. Despite a subtle effect derived from a short term blockage of CLK/CYC function cannot be ruled out, our results suggest that structural remodeling of the LNv terminals is even more sensitive to the alterations in glial physiology than PDF levels themselves.

Glia-to-neuron communication actively participates in the circadian regulation of terminal remodeling despite the mechanisms remain to be uncovered. One possible scenario would depend on circadian release of gliotransmitters, as it has been shown to take place in mammalian astrocytes (i.e., ATP; Burkeen et al., 2011; Marpegan et al., 2011), or other ligands known to mediate neuro-glial communication [obvious candidates to test belong to the Fibroblast growth factor (FGF) and Bone morphogenetic protein BMP signaling pathways; Awasaki et al., 2011; Fuentes-Medel et al., 2012; Stork et al., 2014]. These molecules could alter excitability of the neuronal terminals, ultimately affecting activity-dependent mechanisms known to be required for structural plasticity (Sivachenko et al., 2013), or be more directly involved in the remodeling process.

Circadian structural remodeling has also been described in the mammalian suprachiasmatic nucleus (SCN). Interestingly, antiphasic cyclical changes in glial coverage of VIP and AVP neurons were reported in the rat SCN and were proposed to contribute to synchronization of the clock to the light-dark cycle (Becquet et al., 2008; Girardet et al., 2010). Although, little is known about circadian structural remodeling in the mammalian brain the pervasive conservation of the mechanisms underlying the molecular clock as well as those underlying synaptic plasticity would predict conservation on this phenomenon as well.

## Author contributions

AH, JD, MFC designed experiments. AH, JD performed and analyzed experiments. AH, JD, MFC wrote the manuscript.

### Conflict of interest statement

The authors declare that the research was conducted in the absence of any commercial or financial relationships that could be construed as a potential conflict of interest.

## References

[B1] Appelbaum L. Wang G. Yokogawa T. Skariah G. M. Smith S. J. Mourrain P.. (2010). Circadian and homeostatic regulation of structural synaptic plasticity in hypocretin neurons. Neuron 68, 87–98. 10.1016/j.neuron.2010.09.006 20920793 PMC2969179

[B2] Awasaki T. Huang Y. O'connor M. B. Lee T. (2011). Glia instruct developmental neuronal remodeling through TGF-beta signaling. Nat. Neurosci. 14, 821–823. 10.1038/nn.2833 21685919 PMC3337551

[B3] Bainton R. J. Tsai L. T. Schwabe T. Desalvo M. Gaul U. Heberlein U. (2005). Moody encodes two GPCRs that regulate cocaine behaviors and blood-brain barrier permeability in Drosophila. Cell 123, 145–156. 10.1016/j.cell.2005.07.029 16213219

[B4] Barnes C. A. (2001). Plasticity in the aging central nervous system. Int. Rev. Neurobiol. 45, 339–354. 10.1016/S0074-7742(01)45018-5 11130905

[B5] Becquet D. Girardet C. Guillaumond F. Francois-Bellan A. M. Bosler O. (2008). Ultrastructural plasticity in the rat suprachiasmatic nucleus. Possible involvement in clock entrainment. Glia 56, 294–305. 10.1002/glia.20613 18080293

[B6] Bernardinelli Y. Nikonenko I. Muller D. (2014). Structural plasticity: mechanisms and contribution to developmental psychiatric disorders. Front. Neuroanat. 8:123. 10.3389/fnana.2014.00123 25404897 PMC4217507

[B7] Borycz J. Borycz J. A. Loubani M. Meinertzhagen I. A. (2002). tan and ebony genes regulate a novel pathway for transmitter metabolism at fly photoreceptor terminals. J. Neurosci. 22, 10549–10557. 12486147 10.1523/JNEUROSCI.22-24-10549.2002 PMC6758454

[B8] Bosler O. Girardet C. Franc J. L. Becquet D. Francois-Bellan A. M. (2015). Structural plasticity of the circadian timing system. An overview from flies to mammals. Front. Neuroendocrinol. 38, 50–64. 10.1016/j.yfrne.2015.02.001 25703789

[B9] Bozorgmehr T. Ardiel E. L. Mcewan A. H. Rankin C. H. (2013). Mechanisms of plasticity in a *Caenorhabditis elegans* mechanosensory circuit. Front. Physiol. 4:88. 10.3389/fphys.2013.00088 23986713 PMC3750945

[B10] Burkeen J. F. Womac A. D. Earnest D. J. Zoran M. J. (2011). Mitochondrial calcium signaling mediates rhythmic extracellular ATP accumulation in suprachiasmatic nucleus astrocytes. J. Neurosci. 31, 8432–8440. 10.1523/JNEUROSCI.6576-10.2011 21653847 PMC3125703

[B11] Ceriani M. F. Hogenesch J. B. Yanovsky M. Panda S. Straume M. Kay S. A. (2002). Genome-wide expression analysis in Drosophila reveals genes controlling circadian behavior. J. Neurosci. 22, 9305–9319. 12417656 10.1523/JNEUROSCI.22-21-09305.2002 PMC6758054

[B12] Chaturvedi R. Reddig K. Li H. S. (2014). Long-distance mechanism of neurotransmitter recycling mediated by glial network facilitates visual function in Drosophila. Proc. Natl. Acad. Sci. U.S.A. 111, 2812–2817. 10.1073/pnas.1323714111 24550312 PMC3932938

[B13] Chen W. F. Maguire S. Sowcik M. Luo W. Koh K. Sehgal A. (2015). A neuron-glia interaction involving GABA transaminase contributes to sleep loss in sleepless mutants. Mol. Psychiatry 20, 240–251. 10.1038/mp.2014.11 24637426 PMC4168011

[B14] Damulewicz M. Rosato E. Pyza E. (2013). Circadian regulation of the Na+/K+-ATPase alpha subunit in the visual system is mediated by the pacemaker and by retina photoreceptors in *Drosophila melanogaster*. PLoS ONE 8:e73690 10.1371/journal.pone.0073690 24040028 PMC3769360

[B15] Depetris-Chauvin A. Berni J. Aranovich E. J. Muraro N. I. Beckwith E. J. Ceriani M. F. (2011). Adult-specific electrical silencing of pacemaker neurons uncouples molecular clock from circadian outputs. Curr. Biol. 21, 1783–1793. 10.1016/j.cub.2011.09.027 22018542 PMC3226771

[B16] Depetris-Chauvin A. Fernandez-Gamba A. Gorostiza E. A. Herrero A. Castano E. M. Ceriani M. F. (2014). Mmp1 processing of the PDF neuropeptide regulates circadian structural plasticity of pacemaker neurons. PLoS Genet. 10:e1004700. 10.1371/journal.pgen.1004700 25356918 PMC4214601

[B17] Fernández M. P. Berni J. Ceriani M. F. (2008). Circadian remodeling of neuronal circuits involved in rhythmic behavior. PLoS Biol. 6:e69. 10.1371/journal.pbio.0060069 18366255 PMC2270325

[B18] Frank M. G. Cantera R. (2014). Sleep, clocks, and synaptic plasticity. Trends Neurosci. 37, 491–501. 10.1016/j.tins.2014.06.005 25087980 PMC4152403

[B19] Fuentes-Medel Y. Ashley J. Barria R. Maloney R. Freeman M. Budnik V. (2012). Integration of a retrograde signal during synapse formation by glia-secreted TGF-beta ligand. Curr. Biol. 22, 1831–1838. 10.1016/j.cub.2012.07.063 22959350 PMC3605899

[B20] Girardet C. Becquet D. Blanchard M. P. Francois-Bellan A. M. Bosler O. (2010). Neuroglial and synaptic rearrangements associated with photic entrainment of the circadian clock in the suprachiasmatic nucleus. Eur. J. Neurosci. 32, 2133–2142. 10.1111/j.1460-9568.2010.07520.x 21143667

[B21] Gorostiza E. A. Depetris-Chauvin A. Frenkel L. Pirez N. Ceriani M. F. (2014). Circadian pacemaker neurons change synaptic contacts across the day. Curr. Biol. 24, 2161–2167. 10.1016/j.cub.2014.07.063 25155512 PMC4175170

[B22] Gorska-Andrzejak J. (2013). Glia-related circadian plasticity in the visual system of Diptera. Front. Physiol. 4:36. 10.3389/fphys.2013.00036 23986707 PMC3750947

[B23] Gorska-Andrzejak J. Makuch R. Stefan J. Gorlich A. Semik D. Pyza E. (2013). Circadian expression of the presynaptic active zone protein Bruchpilot in the lamina of *Drosophila melanogaster*. Dev. Neurobiol. 73, 14–26. 10.1002/dneu.22032 22589214

[B24] Holtmaat A. Svoboda K. (2009). Experience-dependent structural synaptic plasticity in the mammalian brain. Nat. Rev. Neurosci. 10, 647–658. 10.1038/nrn2699 19693029

[B25] Im S. H. Li W. Taghert P. H. (2011). PDFR and CRY signaling converge in a subset of clock neurons to modulate the amplitude and phase of circadian behavior in Drosophila. PLoS ONE 6:e18974. 10.1371/journal.pone.0018974 21559487 PMC3084726

[B26] Lear B. C. Zhang L. Allada R. (2009). The neuropeptide PDF acts directly on evening pacemaker neurons to regulate multiple features of circadian behavior. PLoS Biol. 7:e1000154. 10.1371/journal.pbio.1000154 19621061 PMC2702683

[B27] Lerner I. Bartok O. Wolfson V. Menet J. S. Weissbein U. Afik S.. (2015). Clk post-transcriptional control denoises circadian transcription both temporally and spatially. Nat. Commun. 6:7056. 10.1038/ncomms8056 25952406 PMC4915573

[B28] Lin Y. Stormo G. D. Taghert P. H. (2004). The neuropeptide pigment-dispersing factor coordinates pacemaker interactions in the Drosophila circadian system. J. Neurosci. 24, 7951–7957. 10.1523/JNEUROSCI.2370-04.2004 15356209 PMC6729918

[B29] Liu H. Zhou B. Yan W. Lei Z. Zhao X. Zhang K.. (2014). Astrocyte-like glial cells physiologically regulate olfactory processing through the modification of ORN-PN synaptic strength in Drosophila. Eur. J. Neurosci. 40, 2744–2754. 10.1111/ejn.12646 24964821

[B30] Marpegan L. Swanstrom A. E. Chung K. Simon T. Haydon P. G. Khan S. K.. (2011). Circadian regulation of ATP release in astrocytes. J. Neurosci. 31, 8342–8350. 10.1523/JNEUROSCI.6537-10.2011 21653839 PMC3135876

[B31] McGuire S. E. Roman G. Davis R. L. (2004). Gene expression systems in Drosophila: a synthesis of time and space. Trends Genet. 20, 384–391. 10.1016/j.tig.2004.06.012 15262411

[B32] Mehnert K. I. Beramendi A. Elghazali F. Negro P. Kyriacou C. P. Cantera R. (2007). Circadian changes in Drosophila motor terminals. Dev. Neurobiol. 67, 415–421. 10.1002/dneu.20332 17443798

[B33] Mehnert K. I. Cantera R. (2008). A peripheral pacemaker drives the circadian rhythm of synaptic boutons in Drosophila independently of synaptic activity. Cell Tissue Res. 334, 103–109. 10.1007/s00441-008-0670-0 18688648

[B34] Mehnert K. I. Cantera R. (2011). Circadian rhythms in the morphology of neurons in Drosophila. Cell Tissue Res. 344, 381–389. 10.1007/s00441-011-1174-x 21562943

[B35] Melom J. E. Littleton J. T. (2013). Mutation of a NCKX eliminates glial microdomain calcium oscillations and enhances seizure susceptibility. J. Neurosci. 33, 1169–1178. 10.1523/JNEUROSCI.3920-12.2013 23325253 PMC3600868

[B36] Ng F. S. Jackson F. R. (2015). The ROP vesicle release factor is required in adult *Drosophila glia* for normal circadian behavior. Front. Cell. Neurosci. 9:256. 10.3389/fncel.2015.00256 26190976 PMC4490253

[B37] Ng F. S. Tangredi M. M. Jackson F. R. (2011). Glial cells physiologically modulate clock neurons and circadian behavior in a calcium-dependent manner. Curr. Biol. 21, 625–634. 10.1016/j.cub.2011.03.027 21497088 PMC3081987

[B38] Ozkaya O. Rosato E. (2012). The circadian clock of the fly: a neurogenetics journey through time. Adv. Genet. 77, 79–123. 10.1016/B978-0-12-387687-4.00004-0 22902127

[B39] Park J. H. Helfrich-Forster C. Lee G. Liu L. Rosbash M. Hall J. C. (2000). Differential regulation of circadian pacemaker output by separate clock genes in Drosophila. Proc. Natl. Acad. Sci. U.S.A. 97, 3608–3613. 10.1073/pnas.97.7.3608 10725392 PMC16287

[B40] Petsakou A. Sapsis T. P. Blau J. (2015). Circadian rhythms in rho1 activity regulate neuronal plasticity and network hierarchy. Cell 162, 823–835. 10.1016/j.cell.2015.07.010 26234154 PMC4537806

[B41] Pyza E. Gorska-Andrzejak J. (2004). Involvement of glial cells in rhythmic size changes in neurons of the housefly's visual system. J. Neurobiol. 59, 205–215. 10.1002/neu.10307 15085538

[B42] Pyza E. Meinertzhagen I. A. (1999). Daily rhythmic changes of cell size and shape in the first optic neuropil in *Drosophila melanogaster*. J. Neurobiol. 40, 77–88. 10.1002/(SICI)1097-4695(199907)40:1<77::AID-NEU7>3.0.CO;2-0 10398073

[B43] Ruiz S. Ferreiro M. J. Casanova G. Olivera A. Cantera R. (2010). Synaptic vesicles in motor synapses change size and distribution during the day. Synapse 64, 14–19. 10.1002/syn.20699 19725115

[B44] Ruiz S. Ferreiro M. J. Menhert K. I. Casanova G. Olivera A. Cantera R. (2013). Rhythmic changes in synapse numbers in *Drosophila melanogaster* motor terminals. PLoS ONE 8:e67161. 10.1371/journal.pone.0067161 23840613 PMC3695982

[B45] Rutila J. E. Suri V. Le M. So W. V. Rosbash M. Hall J. C. (1998). CYCLE is a second bHLH-PAS clock protein essential for circadian rhythmicity and transcription of Drosophila period and timeless. Cell 93, 805–814. 10.1016/S0092-8674(00)81441-5 9630224

[B46] Seugnet L. Suzuki Y. Donlea J. M. Gottschalk L. Shaw P. J. (2011). Sleep deprivation during early-adult development results in long-lasting learning deficits in adult Drosophila. Sleep 34, 137–146. 10.1093/sleep/34.2.137 21286249 PMC3022932

[B47] Sivachenko A. Li Y. Abruzzi K. C. Rosbash M. (2013). The transcription factor mef2 links the Drosophila core clock to fas2, neuronal morphology, and circadian behavior. Neuron 79, 281–292. 10.1016/j.neuron.2013.05.015 23889933 PMC3859024

[B48] Stork T. Sheehan A. Tasdemir-Yilmaz O. E. Freeman M. R. (2014). Neuron-glia interactions through the Heartless FGF receptor signaling pathway mediate morphogenesis of Drosophila astrocytes. Neuron 83, 388–403. 10.1016/j.neuron.2014.06.026 25033182 PMC4124900

[B49] Stuart A. E. Borycz J. Meinertzhagen I. A. (2007). The dynamics of signaling at the histaminergic photoreceptor synapse of arthropods. Prog. Neurobiol. 82, 202–227. 10.1016/j.pneurobio.2007.03.006 17531368

[B50] Suh J. Jackson F. R. (2007). Drosophila ebony activity is required in glia for the circadian regulation of locomotor activity. Neuron 55, 435–447. 10.1016/j.neuron.2007.06.038 17678856 PMC2034310

[B51] Tanoue S. Krishnan P. Krishnan B. Dryer S. E. Hardin P. E. (2004). Circadian clocks in antennal neurons are necessary and sufficient for olfaction rhythms in Drosophila. Curr.Biol. 14, 638–649. 10.1016/j.cub.2004.04.009 15084278

[B52] Weber P. Kula-Eversole E. Pyza E. (2009). Circadian control of dendrite morphology in the visual system of Drosophila melanogaster. PLoS ONE 4:e4290. 10.1371/journal.pone.0004290 19173003 PMC2628732

[B53] Yoshii T. Wulbeck C. Sehadova H. Veleri S. Bichler D. Stanewsky R.. (2009). The neuropeptide pigment-dispersing factor adjusts period and phase of Drosophila's clock. J. Neurosci. 29, 2597–2610. 10.1523/JNEUROSCI.5439-08.2009 19244536 PMC6666242

