# Supplementary material for: Neuronal and Glial Clocks Underlying Structural Remodeling of Pacemaker Neurons in Drosophila
Source: Front Physiol. 2017 Nov 14;8:918. doi: 10.3389/fphys.2017.00918 (PMC5694478; doi:10.3389/fphys.2017.00918)
Supplement: Supplementary file 1 [file Image1.PDF]

Supplementary Material

Neuronal and glial clocks underlying structural remodeling of pacemaker neurons in *Drosophila*.

Anastasia Herrero, José M. Duhart and M. Fernanda Ceriani

*** Correspondence:** Corresponding Author: fceriani@leloir.org.ar

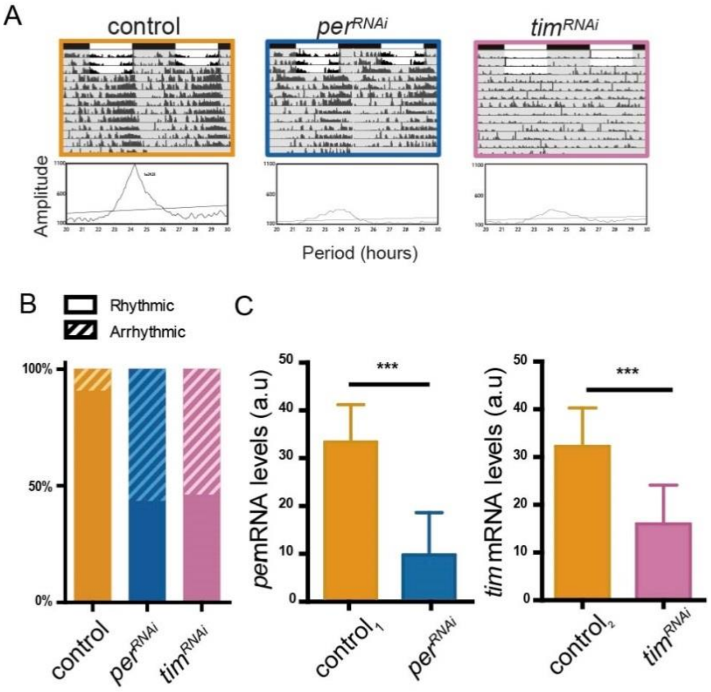

Supplementary Figure 1

Independent RNAis are equally efficient to trigger behavioral phenotypes. (A) Representative double-plotted actograms and their respective periodograms of the different genotypes: UAS-*dcr2* (control), UAS-*per*^{RNAi} (#31285) and UAS-*tim*^{RNAi} (#2886) under *pdf*-Gal4 control. Locomotor activity of individual flies was recorded for 4 days under 12:12 light-dark cycles (LD) and then transferred to constant darkness (DD) (gray area) for 9 additional days. In the actograms, white bars represent day, black bars represent night. For every fly actogram, periodograms of the free-running rhythm in DD are shown. In orange is illustrated the rhythmic behavior of a typical male control fly. In blue and pink, the UAS-*per*^{RNAi} and UAS-*tim*^{RNAi} genotypes respectively, that clearly lost rhythmicity. (B) Percentage of rhythmicity. Data represents at least three independent experiments; over 70 flies were analyzed. (C) Assessment of the transcript levels after RNAi expression under *tim*-Gal4. Levels are normalized to *rpl49*. Both *tim and per* mRNAs are reduced compared from their respective control. Student´s t test showed significant differences in expression levels. Triple asterisks (***) indicates significant differences with $p < 0.001$, N =3.

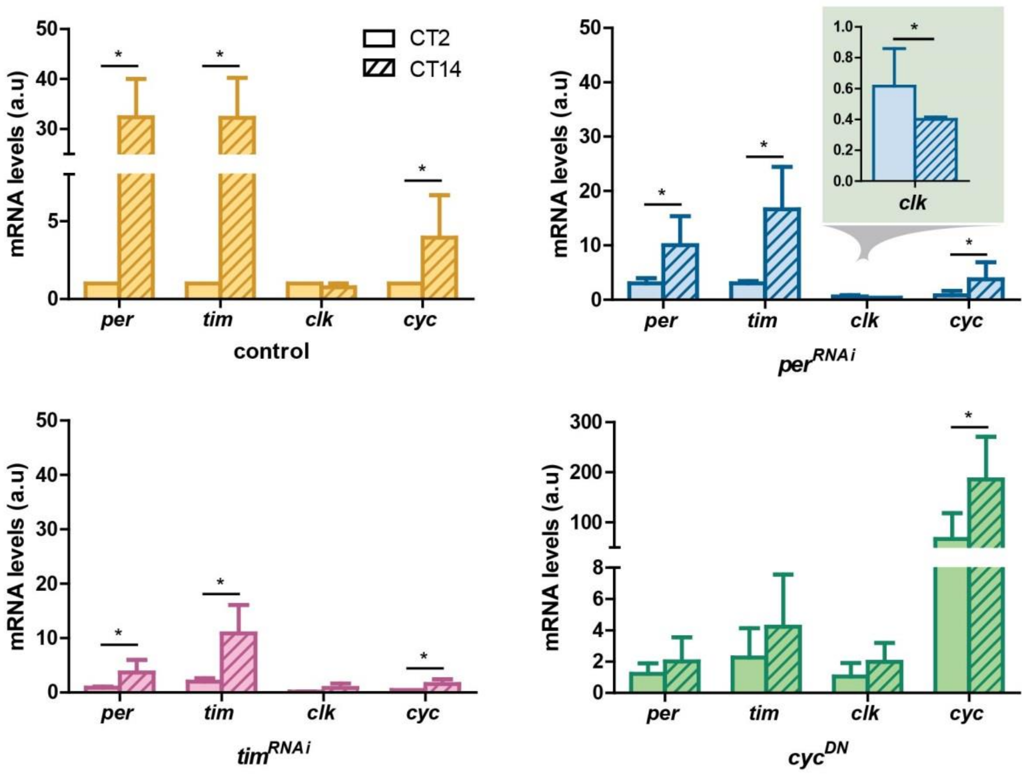

Supplementary Figure 2.

Effects of deregulation of different clock components on molecular oscillations. For each gene mRNA levels were plotted both at CT2 and CT14. Levels were normalized to the reference gene *rpl49*. The genotypes analyzed are as follows: control (orange), *per*^{RNAi}*I* (blue), *tim*^{RNAi}*I* (pink) and *cyc*^{DN} (green), under *tim*-Gal4. Statistical analysis was performed comparing individual transcript levels at the different time points for each gene within genotypes. Student´s t test showed a significant difference in expression levels where indicated. Asterisks (*) indicate significant differences with $p < 0.05$. Three independent experiments were performed. The primers used to detect *cyc* amplified both the endogenous and the expressed construct (hence the increase of *cyc* mRNA levels in the *cyc*^{DN} genotype).

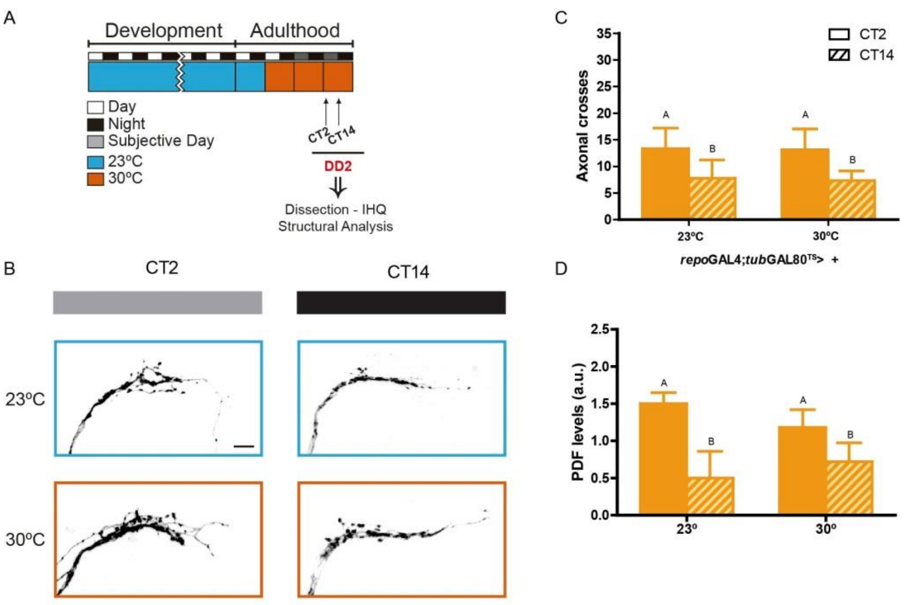

Supplementary Figure 3

Structural plasticity is not affected by temperature. (A) Schematic diagram illustrating the standard protocol. The restrictive condition is shown in light blue (23°C), and the permissive in orange (30°C). (B) Representative confocal images of dsRed immunoreactivity at the dorsal protocerebrum of control flies containing *repo*-Gal4;*tub*-Gal80^{TS}>+, at the early subjective day (CT2, grey bar) and early subjective night (CT14, black bar) during DD2, which is the 3rd day at the permissive condition (30°C). Control flies (raised and maintained at 23°C) are indicated with light-blue. (C) Quantitation of total axonal crosses of *repo*-Gal4;*tub*-Gal80^{TS}>+. Control flies (23°C) display circadian structural remodeling of axonal terminals while animals induced at 30°C show no structural differences. Data represents a single experiment; 10 brains were analyzed per CT/genotype. Different letters indicate statistical differences with a $p<0.05$ (Two-way ANOVA with a Tukey post-hoc test). (D) Quantitation of PDF immunoreactivity at the dorsal protocerebrum at CT2 and CT14 on DD3. Control flies (23°C), exhibit circadian oscillation of PDF levels, and flies at 30°C were not significantly different to control. Same letters indicate no statistically differences ($p>0.05$) (Kruskal-Wallis One-way ANOVA, followed by a Conover post hoc test). Data represents a single experiment; 10 brains per CT/genotype were analyzed.
